# Pediatric naso-sinusal inverted papilloma: report of a case and literature review

**DOI:** 10.11604/pamj.2020.37.373.27186

**Published:** 2020-12-23

**Authors:** Amel El Korbi, Sondes Jellali, Naourez Kolsi, Rachida Bouatay, Leila Njim, Emna Berguaoui, Mehdi Ferjaoui, Khaled Harrathi, Jamel Koubaa

**Affiliations:** 1 Ear-Nose-Throat (ENT) Department, Fattouma Bourguiba University Hospital, Monastir, Tunisia,; 2 Research Unity (UR12SP41), Quality and Security Care, University of Monastir, Monastir, Tunisia,; 3 Anatomical and Cytological Pathology Department, Fattouma Bourguiba University Hospital, Monastir, Tunisia

**Keywords:** Inverted, papilloma, pediatric, endoscopic surgery, case report

## Abstract

Inverted papilloma is a rare nasosinusal tumor that mainly occurs in adults during the 5^th^ decade. The occurrence in children is exceptional and only few cases have been reported in the litterature. Clinical and radiological findings mimic other benign nasosinusal pathologies; therefore, diagnosis is based on histopathology either via biopsy or following surgical excision. Here we present a rare case of pediatric inverted papilloma in a 11-year-old child and we discuss clinical, radiological, therapeutic and evolutionary features through a literature review.

## Introduction

An inverted papilloma (IP) is a rare benign tumor that counts for only 0.5-4% of all nasal neoplasms [1]. IP occurs predominantly in men of the 5^th^-6^th^ decades of life. It is exceptional in the pediatric population and only a handful of cases have been reported in the litterature [2]. IP is known to be a benign tumor, however, it is caractherized by its recurrency particularly if incompletely removed and its malignant transformation possibility [2]. We report in this paper a case of IP in a child dealed with an endoscopic endonasal removal.

## Patient and observation

**Figure 1:**
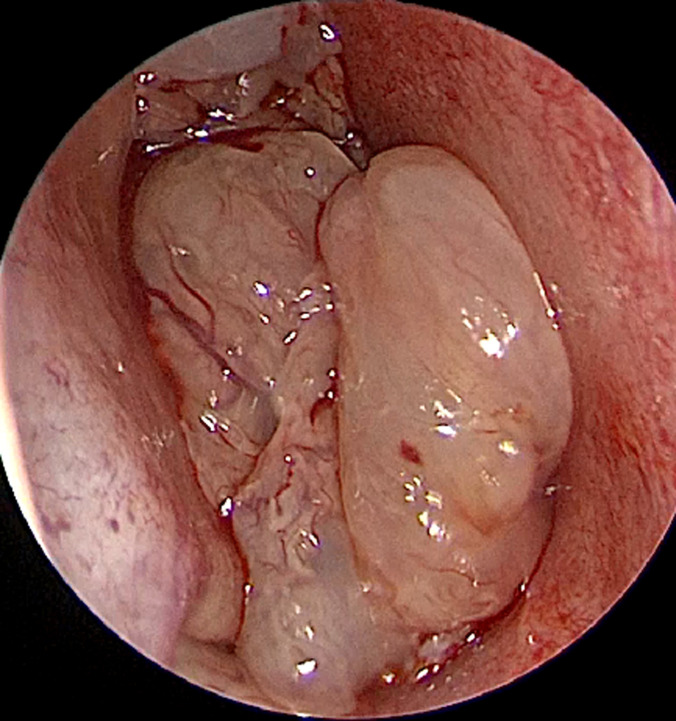
polypoid mass filling the right nasal cavity

**Figure 2:**
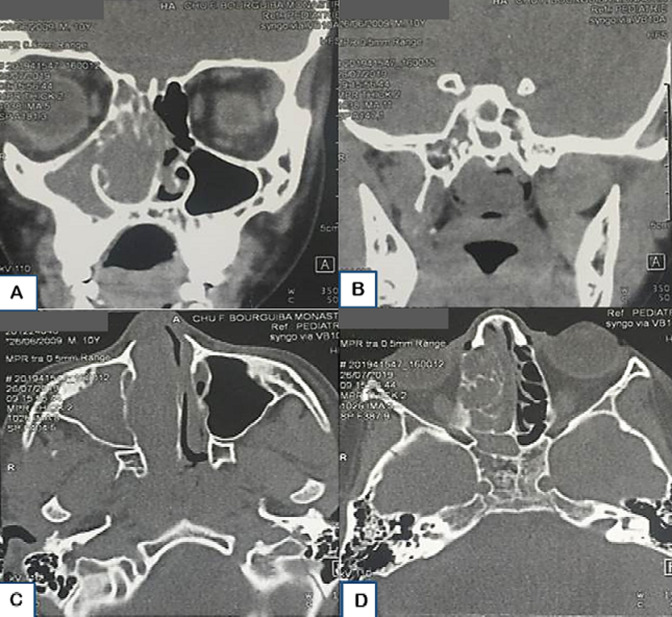
coronal (A, B) and axial (C, D) CT scan showing a large mass of the right nasal cavity with an extension to the choana (A, C) and nasopharynx (B) with a right ethmoidal filling (D)

An 11-year-old boy presented to our department with a 12-month history of right-sided nasal obstruction, purulent nasal discharge and posterior rhinorrhea. His medical, surgical and family history was unremarkable. He had no others rhinological symptoms; particularly no epistaxis nor olfactory disorders. Physical examination with nasal endoscopy found a large polypoid mass (Figure 1) filling completely the right nasal cavity extending to the nasopharynx leading to a left septal deviation. Cervical examination did not show any lymphadenopathy. The ophtalmological and neurological exams were normal. Coronal and axial sinus computerized tomography (CT) revealed a complete filling of the right nasal cavity with choanal, nasopharyngeal and right pansinusal extension associated to a controlateral septal deviation. No bony destruction was observed (Figure 2).

**Figure 3:**
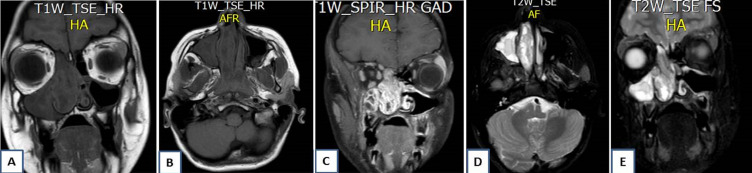
MR images showing an iso-intense T1-weighted sequence right naso-sinusal mass (A, B) with a peripheral enhancing on T1-weighted-gadolinium sequences (C) and “cerebriform” aspect on T2-weighted sequences (D, E)

**Figure 4:**
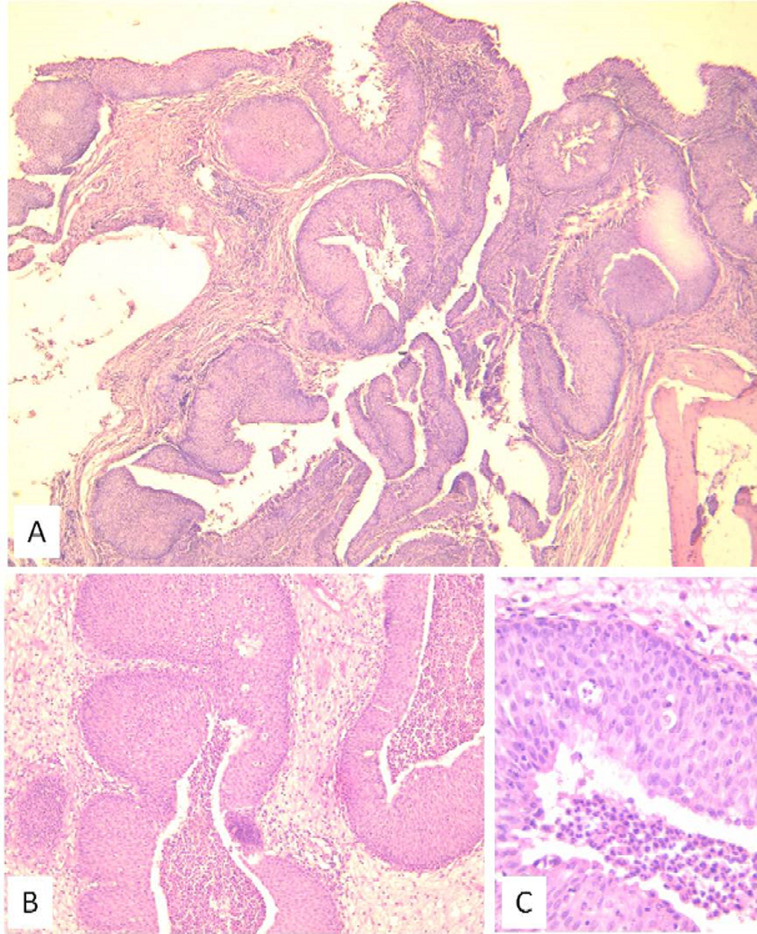
A) (HEx40) endophytic or “inverted” growth pattern consisting of thickened epithelial nests arising from the surface and growing down into the stroma; B) (HEx100) slightly higher magnification showing the endophytic pattern of growth of benign non-keratinizing squamous epithelium; C) (HEx400) the epithelial proliferation is noteworthy of uniformity of nuclei, absence of cytologic atypia and the presence of neutrophils throughout the epithelium

Magnetic resonance imaging (MRI) showed an ethmoido-nasal mass extending lateraly to the maxillary sinus with a meatal enlargement and posteriorly to the nasopharynx. The lesion appeared in heteregenous signal with a cerebriform aspect and a peripheral enhancement on T1-weighted-gadolinium sequences (Figure 3). The diagnosis of inflammatory mass or angiectasic polyp were evoked. Biopsies were performed and histopathological analysis concluded to the diagnosis of an inflammatory polyp. The patient underwent an endonasal endoscopic surgery under general anesthesia. A large medial meatotomy were performed and the IP diagnosis was made on frozen sections of peroperative specimen, thus a right medial maxillectomy and an ethmoidectomy were achieved with a complete removal of the tumor. The patient was discharged two days after. Microscopally, the tumor was made of thickened epithelial nests arising from the surface and growing down into the stroma confiming the enodophytic growth related to the IP (Figure 4). There was no recurrence after six months of follow-up.

## Discussion

Inverted papilloma represents 0.5 to 4% of polypoid lesions of the nose and paranasal sinuses. It characteristically presents in the 5^th^ and 6^th^ decades of life, with a 3: 1 male predilection [1]. IP has rarely been described in the pediatric population. Many large literature studies on inverted papillomas did not report any pediatric case, arguing the paucity of those pathological entities in children and adolescents; Hyam´s landmark study of 149 patients with sinonasal IP included no pediatric cases [3]. Eavey [4], provided the first detailed description in the only published case series of IP in children and adolescents, which included 3 patients aged 6, 9 and 12 years; and two adolescents aged 20 years.

IP in children occurs predominantly in males. Ages range from 6 to 15 years and arise most commonly from the maxillary sinus, nasal septum and also the middle turbinate (Table 1). Our patient was 11 years old. IPs are recognized as benign tumors, with the potential of malignant transformation and recurrence after surgery [2]. In the rare cases reported in children, there has been only one case of malignancy. It was a 20-year-old female who developed low-grade squamous cell carcinoma with an inverted papilloma pattern [4]. Macroscopically IP appear as irregular polypoid masses of variable consistency, pink or gray in color [5]. Histologically, it is characterized by thickened neoplastic epithelium inverted into the underlying connective tissue with an intact basement membrane. The tumor epithelium is composed of well differentiated columnar or ciliated respiratory epithelium with variable squamous differentiation [6].

**Table 1:** pediatric cases of inverted papilloma literature review

	Age (years)	Sex	Locations	Surgery	Follow-up	Recurrence
Stanley (1984)	10	Male	Right inferior turbinate	Caldwell-Luc and ethmoidectomy	2 years	None
Eavey (1985)	6	Male	Right septum	Intranasal removal	Lost	None
	9	Female	Right nasal cavity, maxillary and ethmoid sinuses	Lateral rhinotomy and medial maxillectomy	2½ years	None
	12	Male	Right nose and sinuses	Lateral rhinotomy, medial maxillectomy and sphenoidotomy	2 years	None
Limaye (1989)	15	Female	Left nasal cavity and maxillary sinus	Lateral rhinotomy, ethmoidectomy and antrostomy	3½ years	At 12 months
	10	Male	Inferior portion of the lateral nasal wall the floor of the nose, the inferior half of the septum and the vestibule	Lateral rhinotomy and laser vaporization	13 months	At 10 months
Anthony J D'Angelo (1992)	12	Male	Right nasal cavity and maxillary antrum	Caldwell-Luc procedure with a partial medial maxillectomy	1 years	None
Cooter (1998)	10	Male	Posterior border of the maxillary os, the medial maxillary sinus wall and a portion of the inferior turbinate	Maxillary antrostomy, anterior ethmoidectomy, and partial inferior turbinectomy	1 year	None
Özcan (2005)	9	Female	Left inferior concha and lateral nasal wall	Endoscopic resection and Caldwell-Luc procedure (operated two times for the left intranasal mass)	10 months	None
Cho (2008)	15	Male	Left sphenoid sinuses	Transnasal endoscopic resection	2 years	None
Jayakody (2018)	11	Male	Right nasal cavity and Sinuses (origin: postero-lateral wall of the maxillary sinus)	Transnasal endoscopic resection	5 years	None
Our case (2020)	11	Male	Right nasal cavity, maxillary and ethmoid sinuses	Transnasal endoscopic large medial maxillectomy with ethmoidectmy	6 months	None

Aetiopathogenis of IP remains icompletely understood, however, some etiologic factors such as chronic rhinosinusitis, proliferation of nasal polyps, allergy, environmental pollutants, tobacco and viruses particularly the human papilloma virus (HPV) have been reported in the literature, however, no significant evidence for this theory exists [2]. The signs and symptoms of IP in children are indistinguishable from other unilateral pediatric nasal masses and most commonly include unilateral nasal obstruction, nasal discharge, epistaxis. Headache, diplopia, epiphora or hyposmia suggest invasion into adjacent structures. Further, no single symptom or sign can be statistically linked to carcinoma within a papilloma [7,8]. The cases of IP reported in children showed no substantial differences in its clinical behavior or histologic features compared to IP in adults. Although a significant number of pediatric cases reported were diagnosed after a prior recurrence [4].

Imaging investigations with CT scan and MRI are necessary to analyze nasosinusal masses. In typical cases IP arise from medial wall of nasal cavity (exactly from middle meatal) and extent to maxillary sinus. We can observe intralesional calcifications and/or bone thinning or erosion [2,7]. Maxillary sinus is most commonly involved, but the ethmoidal, frontal and sphenoid sinuses may also be affected. On MRI a convoluted cribriform pattern is highly suggestive of IP [2,9]. In our case, the cerebriform aspect observed on the MRI was suggestive. But the diagnosis of IP was not mentioned by the radiologist and an inflammatory mass or an angiectasic polyp were suspected.

Before surgery, pathologic examination is essential to diagnosis. IP may coexist with an inflammatory process showing inflammatory polyps. This accounts for the false negative rates of up to 17% on biopsy reported in the literature [10]. In our case the analysis of preoperative biopsy showed an iflammatory lesion which was certainly a sentinel polyp. In a case of unilateral polyp, sinonasal tumor, particularly IP, should be always suspected [10]. The locally aggressive behavior and the potential of malignancy of IP explain that the only curative treatment of such tumors in adults is surgery [10-15]. In pediatric population, the treatment seems to be the same as in adults. No particularities in the management approach of pediatric IP have been reported in the several cases of the literature [10-15].

Extra-nasal approach was the “gold standard” to perform the “en bloc” resection of the tumor for about 35 years. Whereas, since the development of the endonasal endoscopic surgery, along the last years, mini-invasive techniques are more and more used. The endoscopic medial maxillectomy and its varieties became the essential way to remove IP [11]. It provides a wide access to specific nasal areas and better visualization of the anatomical stuctures. Compared to open approaches, the endoscopic medial maxillectomy had shown advantages of shorter hospital stay with less complications and similar success rates [12].

Recurrences occurs especially in cases of incomplete resection explained by a poor exposure and visualization of the tumor´s limits. They generally appear within 6 months after surgery, however, cases of IP recurrence have been reported 20 years after in the literature [8]. The recurrence rate of IPs in adults ranges between 14 and 78% [2]. In the pediatric cases, Limaye [13] noted a recurrence in his two cases reported after 10 and 12 months. Özcan [7] reported also, a case of a recurrent IP in a 9-year-old child (Table 1). Endocopic examination at clinical controls is the main way to detect recurrence and thus provide minimal surgery when they are still small-sized [7].

## Conclusion

Although it is exceptional, IP should be evoked as diagnosis of unilateral nasal masses in children. Failure to consider this entity may lead to inadequate treatment and risk of recurrence. Pediatric IP shows clinically, radiologically and pathologically similarities to the adult one thus the diagnosis and treatment approches seem to be the same. Since recurrences can occur after a long period of time, life-long follow-up is required.
